# Grit or Honesty-Humility? New Insights into the Moderating Role of Personality between the Health Impairment Process and Counterproductive Work Behavior

**DOI:** 10.3389/fpsyg.2016.01799

**Published:** 2016-12-01

**Authors:** Andrea Ceschi, Riccardo Sartori, Stephan Dickert, Arianna Costantini

**Affiliations:** ^1^Department of Human Sciences, University of VeronaVerona, Italy; ^2^School of Business and Management, Queen Mary University of LondonLondon, UK

**Keywords:** health impairment process, job demands, exhaustion, counterproductive work behavior, honesty-humility, grit

## Abstract

It is acknowledged that chronic job demands may be depleting workers’ stamina resulting in burnout conditions and ultimately causing further health problems. This relation, known as health impairment process, has recently been considered as a possible explanation for the emergence of counterproductive work behavior (CWB). The present work aims to examine the role of two personality traits (i.e., Grit and Honesty-Humility) in this process. The results, based on a sample of 208 private service sector employees, confirm the presence of a fully mediated process and show how Honesty-Humility positively moderates the relationship between job demands and exhaustion, whereas Grit has a negative effect on the relation between exhaustion and CWB. Implications for assessment procedure and hiring decisions are discussed.

## Introduction

In line with the burnout literature, it is widely assumed that burnout leads to health problems, such as psychosomatic illness, cardiovascular and coronary heart diseases (Ahola et al., 2005; Ahola and Hakanen, 2007; Toppinen-Tanner et al., 2009). Therefore, burnout plays a prominent role in explaining the relationship between rising job demands and an increase in such health problems. In fact, in the last 20 years organizational research has provided evidence of the link between job demands, burnout and health indicators (Brotheridge and Grandey, 2002; Geurts and Sonnentag, 2006; Piko, 2006; Little et al., 2007; Umehara et al., 2007; Dollard and Bakker, 2010; Idris et al., 2012; Schaufeli and Taris, 2014).

**FIGURE 1:**
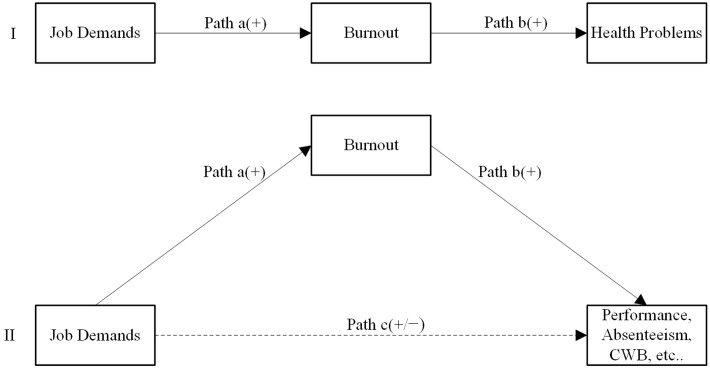
**The original health impairment process (I; Demerouti et al., 2001) and a general extension of the model (II)**.

This two-stage process is known as “*health impairment*” or “*energetic process*,” and it is embedded in the Job Demands Resources (JD-R) model (Demerouti et al., 2001) and empirically supported (Bakker et al., 2004, 2010; Bakker and Demerouti, 2007; Xanthopoulou et al., 2007; Hakanen et al., 2008; Demerouti and Bakker, 2011; Schaufeli and Taris, 2014). Considering research in support of this model, it is likely that the health impairment reflects a more universal process at work, of which the health issue is just one symptom (Balducci et al., 2011). In order to show work-related implications, literature usually provides studies where the health impairment process is associated with outcomes such as organizational well-being or job performance (Bakker et al., 2004, 2008; Idris et al., 2012). The nature of this relationship depends both on the types of job demands as well as on the outcomes considered in the study, resulting in a partial or full mediation through burnout. While job demands are usually negatively related to health through a full and negative mediation with burnout, they are positively and directly associated with task performance. For example, Bakker et al. (2004) found that in-role performance was mostly predicted by job demands through workers’ exhaustion (a component of burnout), while in another study cynicism (another burnout component) predicted teams’ sales performance (Bakker et al., 2008). Based on evidence from research, it is reasonable to assume the presence of two relationships, namely a first path from job demands to burnout (or its components), and a second path from burnout to the outcome considered (e.g., performance, absenteeism, etc.). Finally, we can consider the presence of a third path, when the relation is partially mediated, usually when the outcomes studied are different than health problems (**Figure 1**).

Considering these types of outcomes, the health impairment process has been examined regarding the effect on performance but rarely used to predict behavioral constructs correlated with burnout. In one notable example, in the JD-R framework, job demands predicted burnout and consequently absence duration (Bakker et al., 2003). More recently, some attempts have been made to study the relationships between job demands and organizational behavior in the health impairment process (Schaufeli et al., 2009; Balducci et al., 2011; Nahrgang et al., 2011). Balducci et al. (2011) studied the emergence of counterproductive work behavior (CWB) as a behavioral stress reaction. Workload, role conflict, and interpersonal demands were related to some CWBs via negative affectivity (i.e., anger, pessimism). Finally, a recent study of Smoktunowicz et al. (2015) has confirmed the presence of job burnout as a mediator of the job demands–CWB relationship. The theoretical framework of their study was based on the Demand Control model (DCM; Karasek, 1998). They found that a high level of job demands was indirectly associated with higher CWB frequency, with an increase in job burnout (primarily exhaustion) operating as a mediator.

Counterproductive work behavior can also be predicted by emotional labor (i.e., emotional demands by which workers manage their feelings toward organizational aims; Bechtoldt et al., 2007). This relationship does not include burnout as a mediator so it is not technically definable as a health impairment process, but it reveals the important role of emotional demands in predicting CWB. Moreover, the relationship between emotional labor/acting deep and CWB is moderated by self-control, such that people with high self-control can perform better by effectively dealing with emotional stressors. This highlights the relevance of considering individual dispositions (e.g., self-control) and personality traits in understanding the antecedence of organizational behavior and CWB.

With the exception of the recent study of Smoktunowicz et al. (2015), literature lacks clear research on the moderating impact of individual differences in the two-stage health impairment process and CWB. The current research aims to fill this gap by developing a model that considers the mediated relationship of such a classic process as the health impairment, and having CWB as the final outcome (Path a: job demands → exhaustion; Path b: exhaustion → CWB; and eventually Path c: job demands → CWB). Moreover, this contribution aims at exploring the moderating role performed by two promising personality traits: Honesty-Humility and Grit. Both such traits are relatively new in the organizational domain in comparison with the other well-known big five traits (Sartori et al., 2015, 2016; Ceschi et al., 2016), and have been developed based on different personality theories (Ashton and Lee, 2005; Duckworth et al., 2007). They represent two distinct personality traits which do not overlap, but they may have a different (and explicative) role in moderating the relation between the health impairment process and CWB. Whereas Honesty-Humility, which represents the tendency to be faithful/loyal, has already been shown to negatively moderate the relation between the stressors and CWB (Ashton et al., 2000; Zettler and Hilbig, 2010; Wiltshire et al., 2014; Chirumbolo, 2015), Grit, which predicts success by promoting self-control and goal-persisting at work (Duckworth and Gross, 2014), has rarely been studied in relation to counterproductive behaviors (Littman-Ovadia and Lavy, 2016).

In the next sections, we will first explore CWB as a possible outcome of the health impairment process. Secondly, we will introduce Honesty-Humility and Grit in their relation to the energetic process.

### Counterproductive Work Behavior (CWB): Classical Antecedents and Moderators

Being counterproductive at work involves behavior that is deliberate and opposite to the established interests of the organization (Gruys and Sackett, 2003). It has been described as a range of behaviors that transgress the main organizational norms, to weaken the wellbeing of the organization and threaten the co-workers and customers (Penney and Spector, 2005). Counterproductive behaviors are invasive and destructive phenomena that impair performance and proficiency in the workplace (Spector and Fox, 2005). They have been operationalized in a variety of ways. The most salient form of CWB is physical violence, but it may also take the form of much less stunning behaviors such as gossip, subtle and passive actions, failure to fulfill tasks or pass on information, poor attendance, or intentionally sloppy work (Hershcovis et al., 2007).

The occurrence of CWB has been explained by the stressor-emotion model, in which these behaviors are considered as an emotional reaction to frustration at work, due to a number of environmental stressors that hamper work efficiency (Spector and Fox, 2005). These stressors can be job demands such as interpersonal conflict, workload, emotional labor, role conflict and role ambiguity (Barling et al., 2009). Indeed, if CWB is an outcome of the health impairment process (Balducci et al., 2011), and if the role of such job demands in determining the process is clear, the stressor-emotion model implicitly hides how emotional exhaustion (as a manifestation of frustration) is correlated with CWB. Although the relationship between emotional exhaustion and CWB has been examined less frequently than the relation between job stressors and CWB, there is evidence to sustain such a link. In fact, emotionally exhausted individuals enlist more deviant behavior to relieve antagonistic emotions or for some subservient aim (Banks et al., 2012). Also, emotional exhaustion predicts which employees may be more engaged in CWBs (Krischer et al., 2010; Banks et al., 2012) and self-control seems to buffer this relation (Bolton et al., 2012).

The relation exhaustion → CWB certainly presents some personal factors as reliable moderators, such as self-control (Marcus and Schuler, 2004), but also several personality traits such as narcissism (Penney and Spector, 2002), anger trait or some of the big five traits (Douglas and Martinko, 2001; Marcus et al., 2007; Grijalva and Newman, 2015). For example, agreeableness and conscientiousness are related to interpersonally directed CWBs, and organizationally directed CWBs, respectively. Similarly, extraversion predicted theft at work, whereas openness to experience predicted work deviance.

Aside from the big five model, other moderator traits may better interact with the presented relations. These traits can be found in the new definitions of personality that are consistent with modern theories of development, practices that are informed by high-quality research, and constructs associated with significant challenges (Clement and Bollinger, 2016).

### The Explicative Power of Honesty-Humility in Predicting Unethical Behavior and Workplace Deviance

Considerable research has been conducted within a personality framework alternative to the well-known Big Five. A different personality structure, named the HEXACO model, includes six dimensions instead of five (Ashton et al., 2007). The most salient property of the HEXACO model is the inclusion of the Honesty-Humility dimension. Honesty-Humility measures the disposition to not take advantage of other individuals, even when there is no risk of unfavorable repercussion for such exploitation (Ashton and Lee, 2008). People low in Honesty-Humility are portrayed as egoistic, lying, haughty, fraudulent, unethical, hypocritical and cunning (Lee and Ashton, 2006).

Considering this description, it is not unexpected that low Honesty-Humility is linked with a certain range of questionable behaviors. Low Honesty-Humility individuals have an inclination to deceive, craft, and break rules, searching for the chance to take part in self-interested behaviors (Lee et al., 2005a). The HEXACO Honesty-Humility dimension has consistently predicted workplace deviance and CWBs (Zettler and Hilbig, 2010; O’Boyle et al., 2012). For example, anti-social behaviors toward the organization and co-workers (i.e., workplace vandalism, absenteeism and alcohol abuse at work) were negatively correlated with the Honesty-Humility trait (Lee et al., 2005b).

In relation to the health impairment process, the Honesty-Humility trait has been shown to moderate the effects of a job stressor (i.e., job insecurity) on CWB (Chirumbolo, 2015). Moreover, stressful situational factors, such as the perceived absence of organizational politics, elicit more CWB in individuals with a low score of the Honesty-Humility trait (Wiltshire et al., 2014).

### Grit and the Inclination to Persist in Frustrating Behaviors for Long Term Goals

Scholars have recently proposed a personality construct known as Grit, which represents “*perseverance and passion for long-term goals*” (Duckworth et al., 2007). Gritty individuals have higher educational achievements, at work and in training, gritty professors promote better educational performance of their students, where cadets who show a high grit score are more likely to graduate in an elite military academy (Duckworth et al., 2007; Duckworth and Quinn, 2009; Eskreis-Winkler et al., 2014). Gritty workers outperform their colleagues because they invest more effort in their work, thus allowing people to persevere in tedious and frustrating behaviors (Duckworth et al., 2011). Another important construct protective toward frustrating behaviors is self-control, partially associated to Grit, but distinguished because it focuses on aligning actions and intentions for achieving one’s targets. The importance of self-control in the workplace has been documented in relation to CWB, where this construct moderates the relationship with emotional labor. Moreover, individuals with high self-control are capable of nullifying the effects of depersonalization, organizational misidentification, and passive CWBs (Hirschi and Gottfredson, 2000).

Regarding the relationship with job demands, motivational intensity theory, which is based on a model of how people regulate efforts, provides a natural platform for building predictions about how Grit affects job demands’ perception (Brehm and Self, 1989). Based on this theory, people’s work effort is the sum of the significance given to achievements and the environmental condition for reaching such goals (i.e., team structure, teamwork, etc.). The personal meaning given to success defines how much job demands people are willing to accept to reach their goals. A trait as Grit may affect the inclination to manage job demands toward burnout and its components (Path a) by making the attainment of goals appearing less difficult and stressful (Littman-Ovadia and Lavy, 2016). In contrast to self-control, Grit includes the notion that passion can influence the achievement of goals and also moderate the influence of job demands on perceived exhaustion. For example, a gritty employer might work for extended hours to complete an assignment because he/she feels passionate toward his/her work and does not feel stressed while doing it.

Apart from this evidence, research on Grit in the domain of the classic I/O outcomes is still in its preliminary phase, although some research on the effects of strong character (conceptually close to Grit) on counterproductive behaviors has recently been conducted (Engel, unpublished doctoral dissertation). Considering this premise, Grit seems to be a promising candidate as a moderator of the two relations of the health impairment process.

## Hypotheses

As seen, CWBs are a likely manifestation of the psychological strain in reaction to job demands, such as interpersonal conflict, emotional demands, workload, role conflict, and role ambiguity (Spector and Fox, 2005; Barling et al., 2009). High levels of such job demands are found to be related to the occurrence of CWBs in several studies (Fox et al., 2001; Marcus and Schuler, 2004; Krischer et al., 2010; Grijalva and Newman, 2015). Research demonstrated that the relation between such stressors and CWB can be moderated by traits such as Honesty-Humility (Chirumbolo, 2015). On the other hand, if the role of job demands in determining CWB is clear, the stressor-emotion model conceptually bypasses the mediation of exhaustion as a manifestation of frustration that leads to CWB, and it is not exhaustive in explaining the moderators of this two-stage process. Indeed, exhausted employees have a higher inclination toward CWBs, whereas employees with higher self-control seem to be less sensitive to exhaustion (Marcus and Schuler, 2004). Moreover, Smoktunowicz et al. (2015) has recently shown that high job demands are indirectly related to high CWBs, with job burnout operating as a mediator. In their conclusion, the authors state that “*Future research needs to clarify if the mediating effect of job burnout in the job demands–CWB relationship may be specific for certain components of burnout…*” (p. 345). Certainly, several studies have identified exhaustion as a possible predictor (and mediator) of the emergence of CWB, but its role has never been tested in relation to this two-stage process.

This evidence suggests that the two-stage process of health impairment can be an explicative organizational behavior model for predicting CWB, with exhaustion as a mediator and the personality dimensions of Grit and Honesty-Humility as potential moderators. Personality traits can affect the stress–strain relation in different ways, for instance influencing the reactivity of individuals toward stress perception, as suggested in the reactivity model (Bolger and Zuckerman, 1995). The emergence of CWB is related to the vulnerability model of interaction (Parkes, 1994), where personality is accountable for rendering the individual more or less vulnerable to the effects of stressful events. Recall that in the health impairment process, passion can affect the perception of effort; secondly Grit can also moderate the emergence of CWBs, due to its focus on self-control and the attention for long term goals. Regarding Honesty-Humility, literature has already shown this trait to be one of the strongest predictors and regulators of CWB (Lee et al., 2005a). Less is known instead on the possible interactions between Honesty-Humility and job demands in relation to exhaustion. Competing theories indicate that this relationship could have gone in either direction: it is possible that low Honesty-Humility individuals would be less likely to feel and take care of job demands, thus finding a charged workplace less distressing than high Honesty-Humility individuals; on the other hand, the Honesty-Humility trait is usually negatively related with exhaustion (Wiltshire et al., 2014).

**FIGURE 2:**
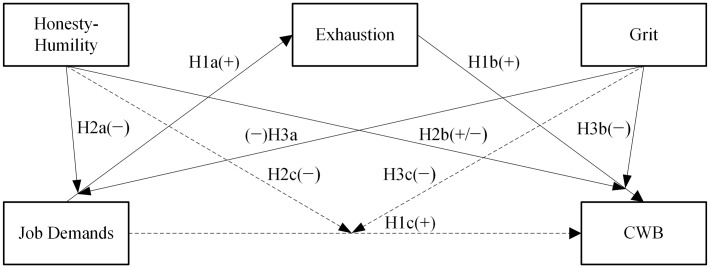
**Hypothesized model of the health impairment process on counterproductive work behavior (CWB) with personality traits as moderators**.

We expect to find a full mediation between: job demands → exhaustion (H1a)→ CWB (H1b), or a partial mediation considering a significant direct effect of job demands on CWB (H1c). We expect a negative moderation effect of Honesty-Humility on the relation between exhaustion → CWB (H2b); if the model is based on a partial mediation, we expect another negative moderation between job demands → CWB (H2c), where for higher levels of exhaustion or of job demands, those high in Honesty-Humility should show lower levels of CWB. We will also explore a possible interaction of Honesty-Humility with job demands → exhaustion (H2a), considering that this relationship could have gone in either directions. Concerning Grit, we expect a negative moderation effect on the relation between job demands → exhaustion (H3a) or/and on exhaustion → CWB (H3b). Specifically, we hypothesize that gritty people’s job demands will less likely lead to exhaustion, and that for these people also exhaustion will less likely lead to CWB. Moreover, if the model is based on partial mediation, we expect another negative moderation between job demands → CWB (H3c; **Figure 2**).

## Materials and Methods

A total of 208 employees operating in the private service sector filled and returned the questionnaire (response rate 80%). Their age ranges between 20 and 60 years with an average of 41 years (*SD* = 9.65). The majority of the sample has higher vocational training (24%) or a high school degree (51%). Most participants work as clerks (63%), 4% are company managers. Most participants (80%) do not supervise staff; only five participants supervise more than five employees. This study was approved by the Ethical Review Committee at the University of Verona. The present sample belongs to a study designed with multiple research purposes.

### Instruments

#### Job Demands

Three specific job demands have been included in the questionnaire: role conflict, emotional demands and hassles. Role conflict has been assessed by using four items derived from the Ivancevich and Matteson (1980) role conflict scale. An example item is “I receive conflicting requests from two or more people” (from 1 = never to 5 = always). Emotional demands are based on a scale developed by Van Veldhoven and Meijman (1994) and include four items. An example is “*Does your work put you in emotional situations*?” (1 = never, 5 = always). Hassles scale (Bakker, 2014) has been used to detect them and it is composed of six items. Examples are: “*I have to deal with administrative hassles”; “I have many hassles to go through to get projects/assignments done”;* (1 = never, 5 = always).

#### Exhaustion

Three exhaustion items of the Oldenburg Burnout Inventory (Demerouti and Bakker, 2008) have been used. Example items are “*There are days when I feel tired before I arrive at work*” and “*After my work, I usually feel worn out and weary*” (1 = totally disagree, 4 = totally agree).

#### CWB

Counterproductive work behavior has been assessed by using the *CWB scale*, i.e., a part of the Individual Work Performance Questionnaire (IWPQ), and we have considered the five CWB items (Koopmans et al., 2012). Example items are: *“I spoke with colleagues about the negative aspects of my work”* and “*I spoke with people from outside the organization about the negative aspects of my work.”* Each item has been rated on a five-point rating scale (0 = never, 4 = very often).

#### Honesty-Humility

Participants have completed the Italian version of the 10-item scale for measuring the *Honesty-Humility* component of the HEXACO-PI-R (Ashton et al., 2006). *“I wouldn’t use flattery to get a raise or promotion at work, even if I thought it would succeed”* and “*I wouldn’t pretend to like someone just to get that person to do favors for me.*” Each item has been rated on a five-point Likert scale (5 = strongly agree; 1 = strongly disagree).

#### Grit

The personality trait of Grit has been assessed by using the Short Grit Scale, an eight-item self-report questionnaire with established construct and predictive validity (Duckworth and Quinn, 2009). Participants have endorsed items by indicating consistency of passions *“I have been obsessed with a certain idea or project for a short time but later lost interest”* (reverse-scored) and consistency of effort “*Setbacks don’t discourage me*,” over time, by using a 5-point Likert-type scale (5 = very much like me, 1 = not at all like me).

## Results

### Descriptive Statistics

**Table 1** shows the means, standard deviations, correlations, and the internal consistency indexes of the scales. All scales present acceptable reliability indexes. In relation to socio-demographic variables, *Honesty-Humility* shows positive correlations with length in service (0.25, *p* < 0.01) and *Grit* instead presents a positive correlation with the number of supervised staff (0.17, *p* < 0.05). A significant and positive correlation is found between most of the job demands measures, exhaustion and CWB, by partially confirming the first hypothesis (H1a; H1b; H1c). Considering possible moderation effects, *Honesty-Humility* and *Grit* showed negative correlations with all the constructs mentioned above. *Honesty-Humility* also shows a positive correlation with length in service, meanwhile *Grit* with the number of collaborators directed. Both traits are positively correlated between them and with the job role (**Table 1**).

**Table 1:** Means, standard deviations (SD), internal consistencies (on the diagonal) and correlations among socio-demographics and study’s variables.

		*M*(*SD*)	1	2	3	4	5	6	7	8	9	10	11	12	13
1	Gender	0.37 (0.48)	-												
2	Age	40.73 (9.65)	-0.18ˆ*	-											
3	Education	3.25 (1.36)	0.06	-0.12	-										
4	Length in service	10.70 (7.12)	0.11	0.42ˆ**	-0.14	-									
5	Number of staff supervised	1.56 (1.28)	-0.14ˆ*	0.06	0.26ˆ**	0.00	-								
6	Job position	1.75 (0.59)	0.04	0.03	0.36ˆ**	0.25ˆ**	0.23ˆ*	-							
7	Role conflict	2.60 (0.72)	-0.11	-0.06	-0.03	-0.19ˆ**	0.14ˆ*	-0.03	(0.75)						
8	Emotional demands	2.42 (0.86)	-0.27ˆ**	0.07	0.18ˆ**	-0.15ˆ*	0.17ˆ*	0.14ˆ*	0.30ˆ**	(0.84)					
9	Hassle	2.62 (0.85)	0.11	-0.20ˆ**	0.17ˆ*	0.10	0.30ˆ**	0.11	0.28ˆ**	0.02	(0.85)				
10	Exhaustion	2.39 (0.58)	-0.03	-0.21ˆ**	-0.12	-0.09	-0.03	-0.14ˆ*	0.26ˆ**	0.17ˆ*	0.11	(0.75)			
11	CWB	0.69 (0.52)	-0.07	-0.10	0.02	-0.20ˆ**	-0.03	-0.12	0.19ˆ**	0.20ˆ**	-0.01	0.40ˆ**	(0.72)		
12	Honesty-Humility	3.91 (0.61)	0.09	0.11	0.10	0.25ˆ**	-0.02	0.17ˆ*	-0.17ˆ*	-0.03	0.01	-0.21ˆ**	-0.26ˆ**	(0.70)	
13	Grit	3.70 (0.54)	0.02	0.02	0.09	0.12	0.17ˆ*	0.18ˆ*	-0.25ˆ**	-0.01	-0.04	-0.17ˆ*	-0.35ˆ**	0.30ˆ**	(0.72)

*Gender: 0 = woman. 1 = men; Education: 1 = Elementary school; 2 = Lower general secondary education; 3 = Higher general secondary education; 4 = Preparatory vocational education; 5 = Higher professional education; 6 = Bachelors’degree; 7 = Masters’ degree; 8 = Ph.D. Length of service: Tenure expressed in years; Employees managed: 1 = Up to 2 supervised co-workers; 2 = 3 to 5 supervised co-workers; 3 = 6 to 10 supervised co-workers; 4 = 11 to 25 supervised co-workers; 5 = More than 25 supervised co-workers; Job position: 1 = Worker; 2 = Senior clerk; 3 = Manager; 4 = Executive; ^∗^*p* < 0.05. ^∗∗^*p* <0.01.*

### Hypotheses Testing

Following the statistical procedure used by Fox et al. (2001) and suggested by Baron and Kenny (1986), we have tested the steps of the health impairment process on CWB (H1a; H1b; H1c). We have also tested the role of Grit and Honesty-Humility at each stage of the process. The single regressions analyses (**Table 2**) have revealed that the presence of exhaustion as a mediator substantially reduces the direct effect of the job demands on CWB, [job demands → CWB: β = 0.18, *p* < 0.01; job demands, (exhaustion) → CWB: β = 0.08*, p* > 0.05]. This invalidates the presence of a partial mediation model (H1c) whilst supporting a full mediation (H1a; H1b); it also makes the analyses of the traits’ moderation effects on the job demands → CWB relationship not relevant to be conducted (H2c; H3c). Regarding the moderation effect of Honesty-Humility, results revealed a significant positive effect on the relation between job demands and exhaustion only (H2a), whereas Grit is a significant negative moderator of the relation between exhaustion and CWB (H3b).

**Table 2:** Regression analyses of moderation effects of Honesty-Humility on Path a, and of Grit on Path b.

Model	Predictors	Exhaustion (Path a)			CWB (Path b)		
		β	*R*^2^	Δ*R*^2^	β	*R*^2^	Δ*R*^2^
Model A	Path × main predictor (PMA)	0.26ˆ**			0.40ˆ**		
			0.07ˆ**	** -**		0.16ˆ**	** -**
Model B	Honesty-Humility	-0.23ˆ**		** -**	-0.26ˆ**		
			0.05ˆ**	** -**		0.07ˆ**	** -**
Model C	Grit	-0.21ˆ**			-0.35ˆ**		
			0.04ˆ**	** -**		0.12ˆ**	** -**
Model A × B	PMA	0.26ˆ**			0.37ˆ**		
	Honesty-Humility	-0.21ˆ**			-0.17ˆ*		
	PMA × Honesty-Humility	0.16ˆ*			-0.07ˆ**		
			0.14ˆ**	0.07		0.20ˆ**	0.04
Model A × C	PMA	0.24ˆ**			0.35ˆ**		
	Grit	-0.18ˆ**			-0.27ˆ**		
	PMA × Grit	0.06			-0.19ˆ**		
			0.10ˆ**	0.03		0.27ˆ**	0.09

*N = 208. Path a main predictor = Job demands; Path b main predictor = Exhaustion; R^2^ = Explanation rate; ΔR^2^ = Change in explanation rate in each step;^∗^p < 0.05. ^∗∗^p < 0.01.*

**FIGURE 3:**
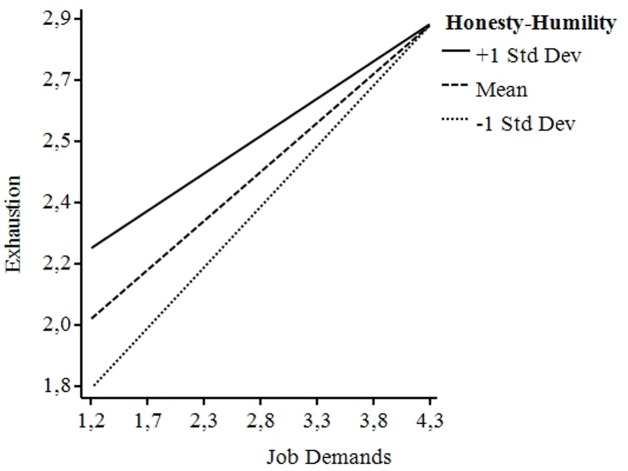
**Graphical representation of the moderation effects of Honesty-Humility on Path a**.

**FIGURE 4:**
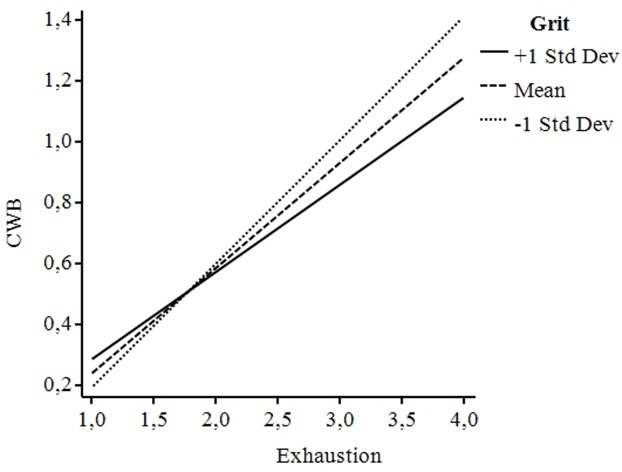
**Graphical representation of the moderation effects of Grit on Path b**.

We next tested the single regression effects found in a comprehensive model, which consists of a full mediation model of exhaustion on job demands-CWB, with the moderations of Honesty-Humility on Path a, and Grit on Path b. We mean-centered and used bootstrapping following the PROCESS procedure recommended by Hayes (2013). The results of these analyses revealed a significant indirect effect of exhaustion 95% CI [0.196,0.474], that fully mediated the effect of job demands on CWB, as revealed by single regressions. Honesty-Humility significantly moderates the effects of job demands on exhaustion, B = 0.16, *p* < 0.05. A simple slope analysis revealed that for lower Honesty-Humility scores, job demands have a stronger positive influence on exhaustion. Data were plotted and the graph (**Figure 3**) revealed that for higher levels of job demands this effect disappears. For Grit, results showed that for lower scores, exhaustion exerts a higher positive influence on CWB, B = -0.18, *p* < 0.05. As for Honesty-Humility, a simple slope analysis was conducted and data were plotted (**Figure 4**). The graph showed that at higher levels of exhaustion the effect of Grit is particularly relevant and robust, such that for those scoring low on Grit, exhaustion has a stronger influence on CWB.

The simple slope analyses revealed that the interactions of both personality traits were significant for all the levels of the moderators (+∖– 1SD), moreover the moderator effects follow an incremental pattern.

## Discussion

The current results contribute to the job stress literature by providing evidence for the potential applicability of the health impairment process outside the area of health research. The present study satisfies the need for clarification regarding the mediating effect of certain components of burnout (such as exhaustion) in the job demands–CWB relationship. Indeed, as suggested by Balducci et al. (2011), it seems likely that the health impairment process reflects an underlying mechanism of human functioning at work. Specifically, we have found that the health impairment process postulated by the JD-R model (Demerouti et al., 2001) also emerges in relation to CWB. Findings indicate that exhaustion mediates the job demands–CWB relationship. High levels of job demands are indirectly associated with higher CWB frequency, with an increase in exhaustion operating as mediator.

Spector and Fox (2005) suggested that CWB may be a reaction to frustration at work due by job demands, and proposed that frustrations are caused by environmental stressors. Considering this evidence and the recent work of Smoktunowicz et al. (2015), it is more plausible to consider the stressor-emotion model as a two-stage process, fairly associable with the health impairment. This is in line with Bolton et al. (2012), who have showed how exhausted employees could have a general higher propensity to engage in CWB.

**FIGURE 5:**
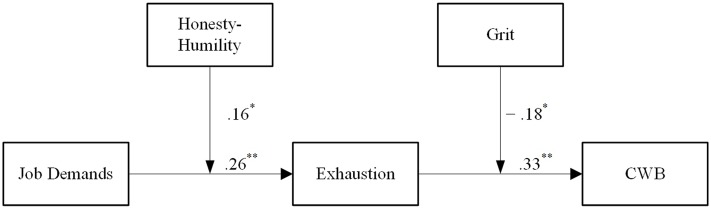
**Model of the health impairment process on CWB with personality traits as moderators**.

These findings contribute to the knowledge on mediating mechanisms, explaining the associations between job demands and CWB. The mediating function of exhaustion can be attributed to the reduction of productive behaviors, so that employees may be more likely to use their working hours for other kinds of behaviors, such as CWB (Smoktunowicz et al., 2015). Concerning the moderating traits, we confirm that Honesty-Humility affects the stressor–strain relation by influencing the reactivity of individuals toward stress perception in relation to job demands, as suggested in the reactivity model (Bolger and Zuckerman, 1995). The emergence of CWB is instead more related to the vulnerability model of interaction of Parkes (1994), where the personality trait of Grit is accountable for rendering the individual less vulnerable to the effects of stressful events (**Figure 5**).

### The Moderation Effects of the Two Traits on the Health Impairment Process

The moderations show that for higher Honesty-Humility scores, job demands have a stronger positive impact on exhaustion, while this effect vanishes for lower levels of Honesty-Humility. Individuals with low scores of Honesty-Humility would be less inclined to feel and take care of job demands, finding a charged work environment less distressing than the individuals with high Honesty-Humility scores. Lower levels of Honesty-Humility are associated with egoistic and deceitful attitudes. Behaviorally speaking, a person with high Honesty-Humility cooperates with others even when one might take advantage of her/him (e.g., by giving too much workload). This disposition to help others is not present in people with low Honesty-Humility. Therefore, they are less likely to experience exhaustion. Literature lacks studies that compare the impact of the Honesty-Humility trait on job stress. In the study of Wiltshire et al. (2014), simple slope analyses revealed that the relation between the perceptions of organizational politics and job stress was more robust at lower levels of Honesty-Humility. It is conceivable that those low in Honesty-Humility would be less inclined to perceive the negative effects of organizational politics, for example by finding a politically charged work environment less unpleasant than those high in Honesty-Humility because of their greater disposition to use maneuvers to prosper in that workplace. Regarding the moderation between Honesty-Humility and CWB, literature has widely shown this trait to be one of the strongest predictors and this strong relation could have hidden the indirect effect researched (Lee et al., 2005b).

Grit represents perseverance and passion for long-term goals and in this definition lies the sense about how Grit could moderate CWB, mainly because of its focus on developing self-control for long term aims (Duckworth et al., 2007). Grit and strength of character include a wide range of attributes: The specific strength impact on individuals’ efficiency at work, mostly related with certain challenging professions (Lavy and Littman-Ovadia, 2016). Grit workers not only dedicate more stamina in a particular task at a given time, but they do it with permanent strength over the years for seeking their long-term goal (Duckworth et al., 2007). For this reason, the relevance accredited to the work domain by highly passionate workers is the capacity of “*run a marathon*” (Littman-Ovadia and Lavy, 2016) and to avoid CWB, which is the major expression of career short-sightedness.

Certainly, most of the research on Grit is longitudinal for this reason, and this is also the most important limitation of the current research. However, evidence from longitudinal studies in the work stress area clearly shows that organizational demands such as workload, role conflict, and hassles have causal consequence on outcomes such as exhaustion and, ultimately, CWB (Balducci et al., 2011; Smoktunowicz et al., 2015). For this reason, the direction of the relations examined is plausible. Nevertheless, longitudinal data is required for a solid proof of the results found, especially in relation to the Grit interaction effect on CWB. Future research should also consider a more representative sample of workers, considering also careers particularly exposed to exhaustion (Di Fabio, 2014; Di Fabio and Kenny, 2015).

A direct practical implication of the current research is the interesting question whether organizations should hire honest and humble or gritty workers. While honesty and perseverance are traits that are probably universally considered to be positive qualities in people, our research suggests that at least Honesty-Humility can have negative consequences for workers’ burnout. It should be noted, however, that Honesty-Humility is positively correlated with length of service, such that people who are more honest also stay longer in their companies. Grit is also positively correlated with the number of supervised staff, which indicates that perseverance may be an important leadership component. Finally, both personality traits tend to co-occur (i.e., are positively correlated) and are related to workers’ job role. This means that the combination of both traits might be particularly suitable for management positions.

## Author Contributions

All authors listed, have made substantial, direct and intellectual contribution to the work, and approved it for publication.

## Conflict of Interest Statement

The authors declare that the research was conducted in the absence of any commercial or financial relationships that could be construed as a potential conflict of interest.
